# Capillaroscopic Evidence of Microvascular Damage in Volleyball Players

**DOI:** 10.3390/ijerph182010601

**Published:** 2021-10-10

**Authors:** Maria Maddalena Sirufo, Alessandra Catalogna, Martina Raggiunti, Francesca De Pietro, Giovanni Galeoto, Enrica Maria Bassino, Lia Ginaldi, Massimo De Martinis

**Affiliations:** 1 Department of Life, Health and Environmental Sciences, University of L’Aquila, 67100 L’Aquila, Italy; maddalena.sirufo@gmail.com (M.M.S.); alessandra.cat4@gmail.com (A.C.); martinaraggiunti@libero.it (M.R.); fra722@hotmail.it (F.D.P.); enricamaria.bassino@gmail.com (E.M.B.); lia.ginaldi@cc.univaq.it (L.G.); 2 Allergy and Clinical Immunology Unit, Center for the diagnosis and treatment of Osteoporosis, AUSL 04 Teramo, 64100 Teramo, Italy; 3 Department of Human Neurosciences Sapienza, University of Rome, 00185 Rome, Italy; giovanni.galeoto@uniroma1.it; 4 IRCCS Neuromed, 86077 Pozzilli, Italy

**Keywords:** volleyball, volleyball player, Raynaud’s phenomenon, microvascular dysfunction, microcirculation, nailfold video capillaroscopy, endothelial dysfunction

## Abstract

Volleyball players experience repetitive stress that involves their hands and, in particular, their fingers. Literature reports that repetitive trauma can lead to local vascular abnormalities, such as reduced capillarization and lower resting blood flow. These anomalies could be related to the presence of dysfunctional endothelium. The aim of this study is to correlate the capillaroscopic findings by nailfold video capillaroscopy (NVC) to volleyball practice in order to early detect possible anomalies and perform an adequate follow-up to avoid damages that could negatively affect sport practice and the players’ health status. In this study, 38 subjects were enrolled, 19 volleyball players and 19 healthy non-players as a comparison group. In almost all the players, we found capillaroscopic alterations of the “aspecific pattern” type without substantial gender differences. We may assume that the repeated traumas involving players’ fingers can negatively modify their microcirculation. Based on these observations, it could be a desirable clinical practice to screen professional volleyball players with NVC in order to implement preventive strategies aimed at protecting the health of athletes.

## 1. Introduction

Volleyball is one of the most popular sports worldwide, with a large growth in the number of players over the last two decades. This sport has been included as a medal sport in the Olympic games since 1959, according to the Fédération Internationale de Volleyball [1]. Volleyball has many health benefits, including cardiovascular benefits, full arm training, abdominal, leg and buttocks toning, stress reduction, and improved hand-eye coordination. However, it is also associated with an increased risk of acute or chronic vascular damage and musculoskeletal injuries [2,3].

Volleyball players experience repetitive stress to hands and, in particular, to fingers. Literature reports that repetitive injury can lead to local abnormalities in the vasculature, such as reduced capillarization and lower resting blood flow [4,5]. The cumulative sport-specific exposure might contribute to the onset of ischemia-related symptoms [6].

It has been demonstrated that after a period of absent or decreased flow (total or partial ischemia), the tissue paradoxically can be damaged by restoration of blood flow (reperfusion), resulting in microvascular dysfunction [7,8]. An impaired blood flow and vascularization might be related to the presence of endothelial dysfunction, the inner layer of cells in blood vessels [9]. The endothelial cells (ECs) have an important role in the regulation of blood flow because they provide an active antithrombotic surface through the production of endotoxins able to balance vasoconstriction and vasodilation and ensure endothelial homeostasis in normal conditions. Alterations, such as inflammation or intense hydrodynamic shear stress, can dysregulate this protective system, promoting a prothrombotic and antifibrinolytic microenvironment [10,11]. In particular, it was observed that after ischemia/reperfusion, ECs could suffer from oxidative stress detaching from the basement membrane, especially in postcapillary venules [12], leukocytes adhere and transmigrate, and vascular permeability increases [13,14,15].

Therefore, it can be hypothesized that volleyball players may be more subject than the general population to dysfunction of the microcirculation because of repetitive trauma to fingers as commonly happens in patients with hand-arm vibration syndrome (HAVS), a secondary form of RP [16] characterized by cutaneous microvascular dysfunction referable to impaired endothelium-dependent vasodilatation resulting in reduced fingers blood flow [17,18].

It is well known that acute inflammation can occur in hands and fingers after intense use of vibration devices, in particular, HAVS are described in chronic vascular symptoms such as vasospasm and blanching of fingers [18]. Exposure to hand-held vibrating tools may cause endothelial injury, in particular, a study conducted on human endothelial cells underlines that vibrations can modulate the phosphorylation of the extracellular signal-regulated kinase (ERK1/2). Given that ERK1/2 can act as a pro-inflammatory pathway, this may account for the acute in vivo inflammation [19,20].

The gold standard for the study of microcirculation is NVC, a non-invasive imaging tool [21] that identifies three capillaroscopic patterns: normal, non-specific, and scleroderma pattern (early, active, and late) [22]. Recently, we reported a case of a 17-year-old woman volleyball player with swollen and sweating hands, predominantly in the right hand. She performed NCV that showed the presence of “non-specific abnormal pattern” with a lack of morphological homogeneity in the capillaries, the presence of efferent tract ectasia of the loops, apical ectasia, tortuosity and newly formed capillaries with a bushy, branching, and “candelabrum” appearance. This was the first report describing the microvascular abnormalities in a volleyball player [23], and for this reason, we decided to expand the study to a group of volleyball players. The hypothesis of the study is that the microvascular changes seen in volleyball players resemble the changes seen in patients with HAVS. Proposing an NCV in these patients could be useful to early detect possible capillaroscopic anomalies that could be linked to further sensitive, muscular, or vascular disorders and perform an adequate follow-up in order to avoid damages to their sports practice and health status.

## 2. Materials and Methods

### 2.1. Participants

Thirty-eight subjects were enrolled in the study, among those of a local team who expressed their willingness to participate in the study. Subjects with smoking, drug, or alcohol habits, taking medical therapies, or with previous radiation exposure were excluded from the study. Moreover, the enrolled participants did not show signs or symptoms attributable to primary Raynaud’s phenomenon in order to link any capillaroscopic alterations found in the study only to the repetitive volleyball traumas. Physically injured fingers and previous repetitive vibrations exposure precluded participation in the study. All subjects underwent appropriate medical history, physical examination, and blood tests which included antinuclear antibodies (ANA), extractable nuclear antigen (ENA) screening (anti-Sm, RNP, Ro60, Ro62, SS-B, SCl-70, Jo-1), anti-neutrophil cytoplasm antibodies (ANCA), anti-cardiolipin antibodies (ACA), anti-β2-glycoprotein antibodies, anti-double stranded DNA (Anti-dsDNA) antibodies, Rheumatoid Factor and anti-cyclic citrullinated peptide (anti-CCP) antibodies to exclude autoimmunity patterns or rheumatological diseases.

This work was conducted after receiving the patients’ informed consent to participate in the study and to publish this report, in compliance with the ethical standards in the field and the norms established by the Internal Review Board of University of L’Aquila (Comitato Etico di Ateneo D.R. n. 206/2013 and D.R. n. 46/2017).

### 2.2. Measurements

Subjects underwent NVC performed using a probe equipped with 200× magnification (VideoCap software 3.0, DS Medica, Milan, Italy). The NVC was performed in a room at a temperature between 20–25 °C, preceded by acclimatization of the patient for 15–20 min. In order to improve the image resolution, a drop of vegetable oil was placed on the nailfold of each finger before observation [24]. The patients had to abstain from caffeine in the 4–6 h prior to the examination and they were instructed not to remove their fingernail cuticles in the previous month and to avoid microtraumas that could question the results of the examination.

We analyzed capillary density (normally between 9 and 14 homogenously distributed hairpin-shaped capillaries in 1 mm), morphology, dimensions of capillaries, and presence of hemorrhages [23]. We also evaluated the presence of enlarged (i.e., irregular or homogeneous increase of capillary diameter >20 and <50 mm) and giant capillaries (homogeneously enlarged loop with a diameter >50 mm). The possible capillaroscopic pictures are described in Table 1.

The characteristics were analyzed according to the classification proposed by Cutolo et al., which is currently one of the most used to stage the damage of the microcirculation not only in scleroderma [22].

The main parameters considered to analyse the patterns were density and dimension (µm) of capillaries, presence of abnormal morphology and haemorrhages. In a normal condition density is ≥7 capillaries/mm, dimension of capillaries is ≤20 µm, absence of haemorrhages and the capillaries have a convex head and hairpin, crossing or tortuous shape. Variations from these morphological aspects are considered anomalies. Aspecific pattern is characterized by the presence of at least one or combination of the following characteristics, decreased capillary density, sizes between 20 and 50 µm, presence of morphological abnormalities and/or haemorrhages. In early scleroderma pattern density of capillaries is preserved, dimensions are ≥50 µm, there no abnormal morphology with present or not of haemorrhages [25].

### 2.3. Statistical Analysis

**Figure 1:**
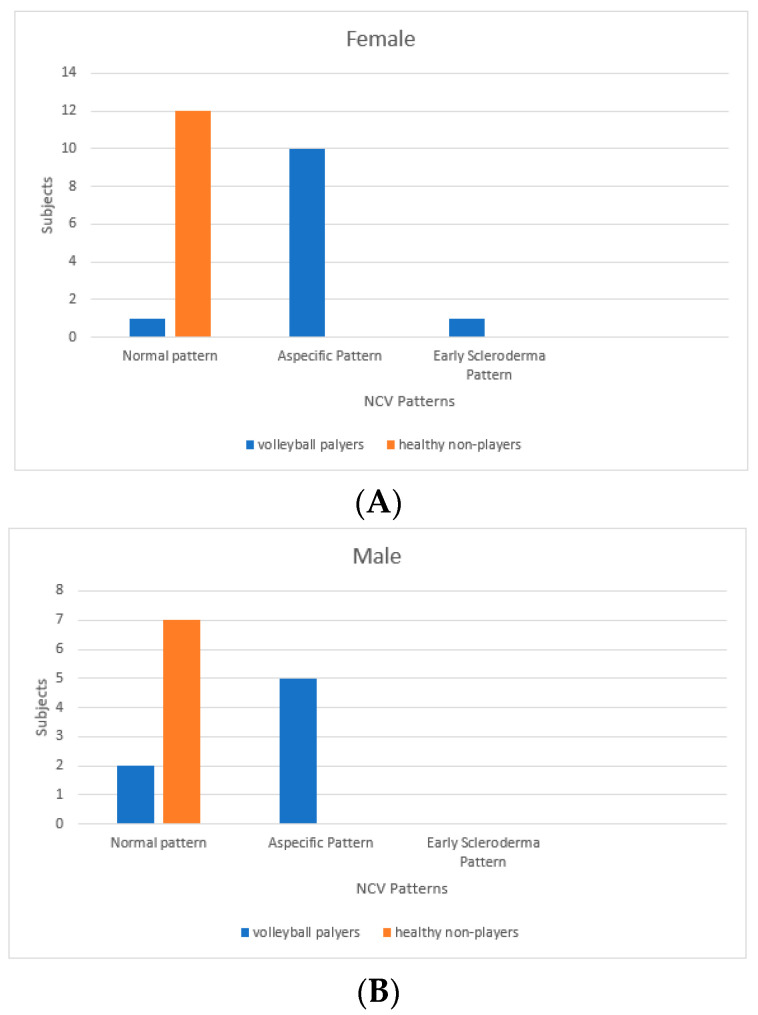
(**A**) Female subjects divided by nailfold video-capillaroscopic (NVC)patterns; (**B**) Male subjects divided by nailfold video-capillaroscopic (NVC) patterns.

Mean, median and standard deviation were used for the descriptive analysis of the sample. The Mann-Whitney U Test for independent samples was used to assess the differences between the groups [26,27]. Statistical significance was set at 0.05. SPSS version 27 software (IBM Corporation New Orchard Road Armonk, NY 10504 USA, was used to carry out the analyses (Figure 1).

## 3. Results

**Figure 2:**
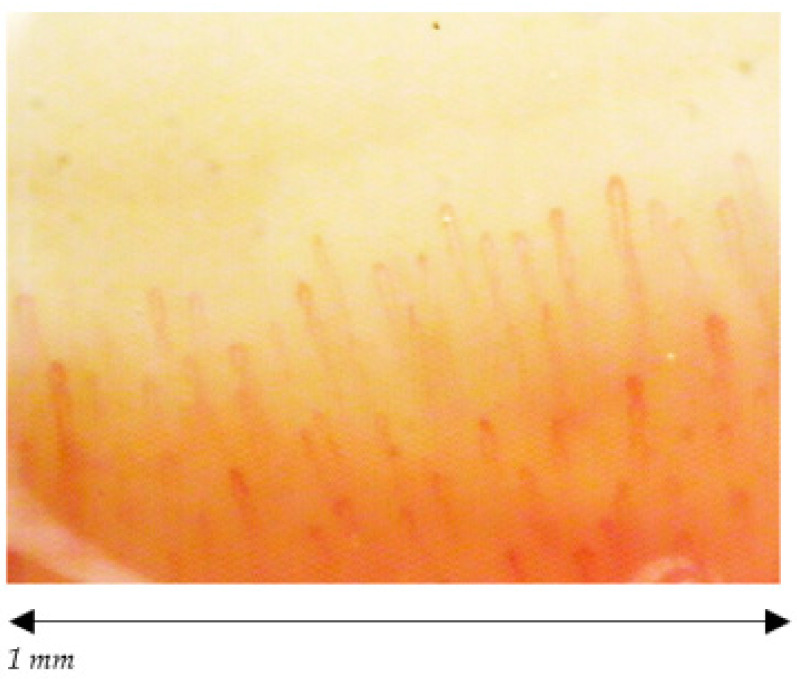
Male normal pattern; density ≥7, normal dimension of capillaries, no abnormal morphology, no hemorrhages.

**Figure 3:**
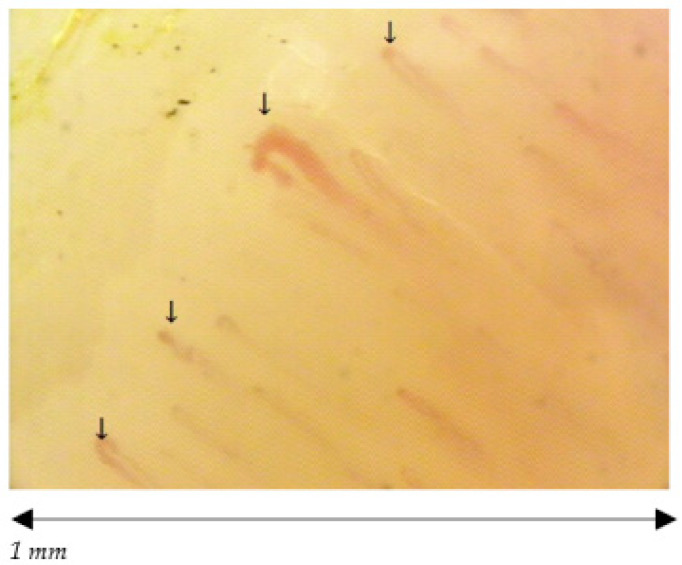
Male aspecific pattern; reduced density of capillaries (↓).

The 38 participants are divided in 19 volley ball players, of which 12 females (27.25 ± 2.86 years old) and 7 males (23.86 ± 1.95 years old) and 19 healthy non-player controls, of which 12 females (25.50 ± 3.06 years old) and 7 males (23.57 ± 2.22 years old). The median and the mode are respectively 26 and 26 for volleyball players and 25 and 21 for healthy non-players controls. We found a statistically significant difference among the capillaroscopic pattern of male players and non-players (*p* = 0.026) (Figure 2 and Figure 3). In five out of seven male volleyball players, the NCV showed an aspecific capillaroscopic pattern, while the pattern was normal in the entire control group.

**Figure 4:**
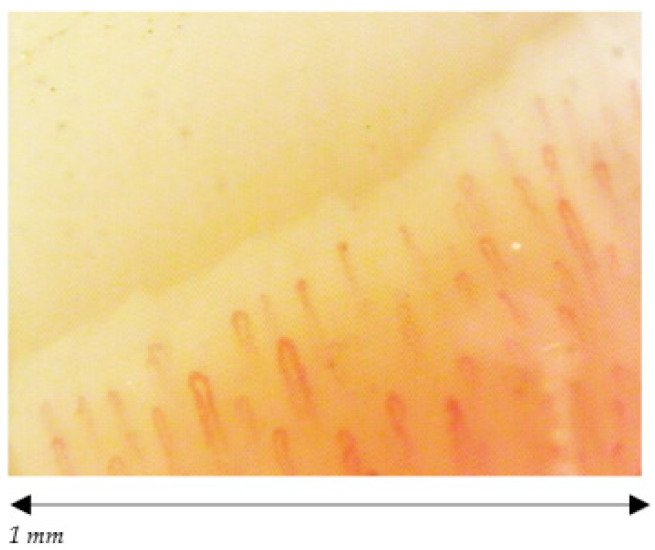
Female normal pattern; normal dimension of capillaries, no abnormal morphology, no hemorrhages.

**Figure 5:**
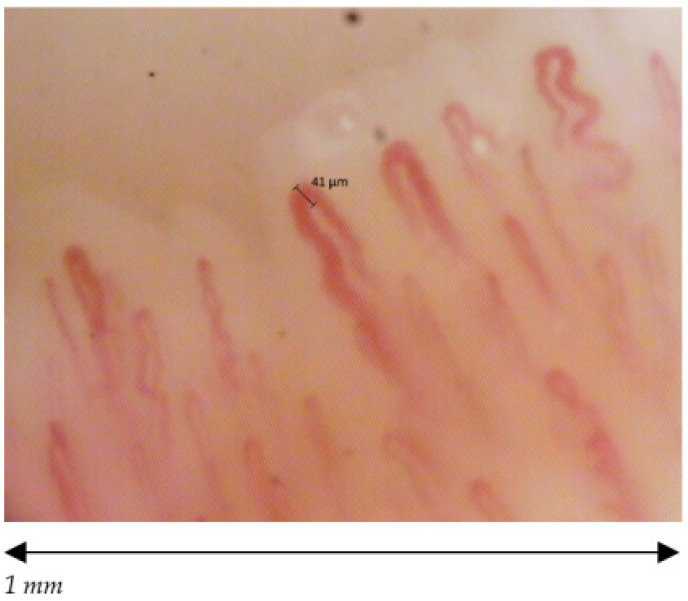
Female aspecific pattern; dimension of capillaries between 20 and 50 µm.

**Figure 6:**
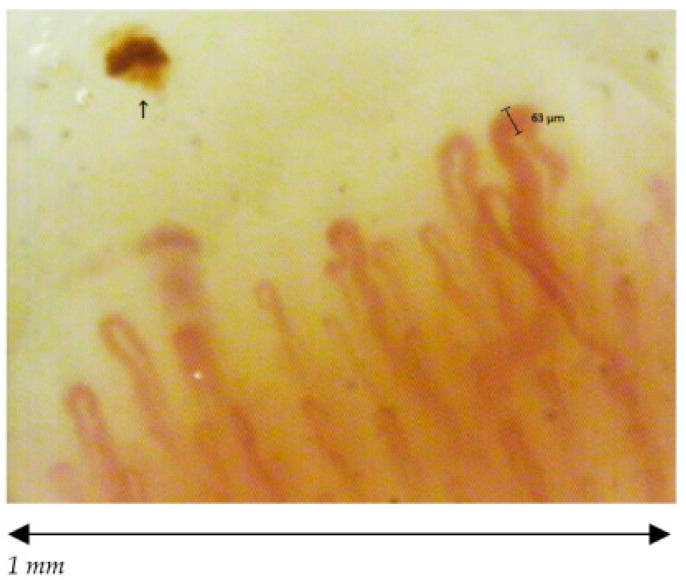
Female early scleroderma pattern; Giant capillary (>50 µ) and hemorrhage (↑).

Furthermore, we found a statistically significant difference among the capillaroscopic pattern of female volleyball players and non- players (*p*< 0.01) (Figure 4, Figure 5 and Figure 6).

Among female players, NCV showed only one case of normal pattern, ten subjects with aspecific pattern and one case of early scleroderma pattern while the pattern was normal in the entire control group. A lack of morphological homogeneity of capillaries, presence ofhaemorrhages, increase in the size and alterations in the flow of the capillaries were observed with greater frequency in those who have played for the longest time and in relation to the role played, especially in people who played as Middle Hitters.Subjects characteristics and capillaroscopic findings are reported in Table 2.

## 4. Discussion

Although limitations related to sample scarcity and disparity between the populations under examination, the study documented significant capillaroscopic differences between volleyball players and healthy controls, both males and females. Training for long time was associated with a greater frequency of capillaroscopic alterations. Microvascular damage can be easily detected by NVC: capillaroscopic changes have been described in athletes [23,28], musicians [29,30], subjects exposed to radiation [31] however “non-specific pattern” (by qualitative assessment) and non-specific capillaroscopic abnormalities (by quantitative assessment) are even found in healthy subjects [25]. Athletes who are exposed to repetitive motion or high-speed collisions may have arterial or venous injuries [32,33]. In particular, volleyball players are often exposed to finger trauma due to the high use of the fingers tips during the game, reaching and hitting the ball.

**Figure 7:**
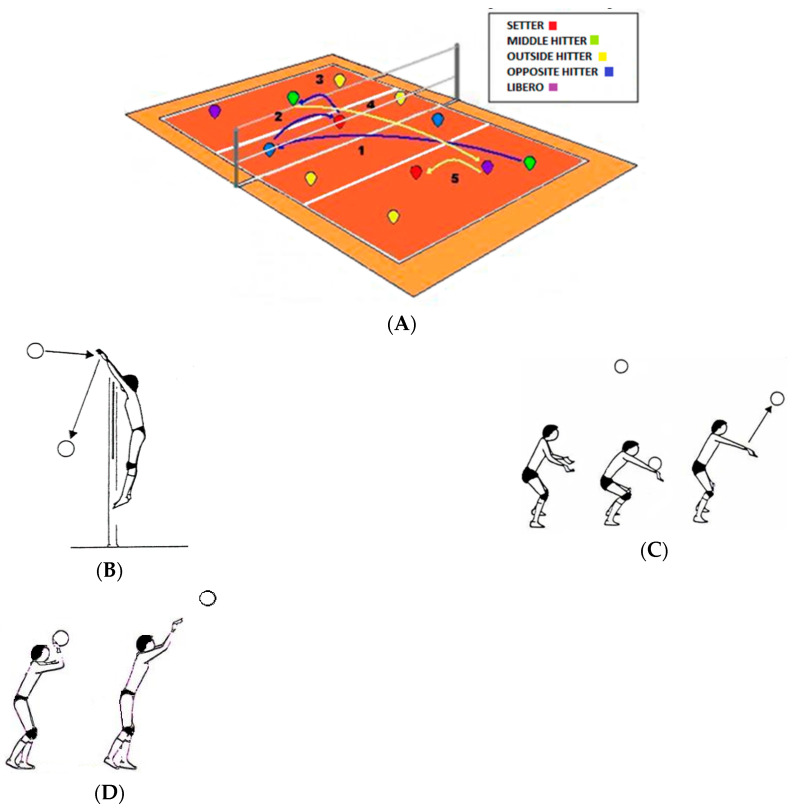

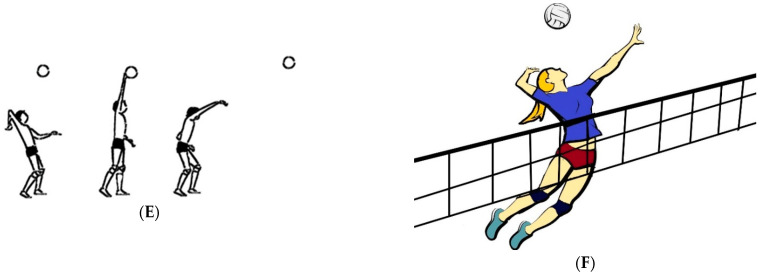
(**A**) Volleyball court with basic game dynamics; (**B**) The middle hitter; (**C**) The libero; (**D**) The setter; (**E**,**F**) The Opposite and Outside hitters.

Volleyball is a team sport that takes place between two teams of six players per team. There are five positions: setter, outside hitter/left side hitter, middle hitter, opposite hitter/right side hitter and libero/defensive specialist, each with a specific key role in the volleyball match [34]. The liberos receive the ball with the bagher on close and supinated forearms to form a large and homogeneous surface. The action performed by the setter is the pass, carried out by bringing the hands over the head, so that the thumbs and index fingers form a figure similar to an inverted heart. The middle hitters mainly act with the block, a jump with arms stretched upwards and hands forming a plane of rejection of the ball towards the opponent’s field. The Opposite and Outside hitters have the task to attack with a blow carried out with concave hand, to give a rotating effect to the ball [32] (Figure 7).

This study documented capillaroscopic alterations in volleyball players. The microcirculation can be damaged due to repetitive trauma resulting in easier and more frequent vasospasm in digital capillaries. [35] In this game, low and high-speed collisions occur between player and ball, these characteristics are different and unique depending on the role played. The incident velocity and the ball type determine the interaction between the ball and the player. Several factors such as kinetic force, intensity, duration and frequency of exposure regulate dangerousness of collisions especially in athletes. Studying different combinations of peak impact force and lost kinetic energy could help to understand the precise role of each of these variables in collision injuries [23]. It could be speculated that the kinetics of collision in relation to the role played and the persistence over time of trauma on the fingers may account for greater frequency of capillaroscopic anomalies in those who have been playing for the longest time. This would be especially true for middle hitters who receive the ball in the most traumatic way. The alteration found in volleyball players are similar to those present in people with HAVS, whose pathogenesis is still incompletely known but it is established that it can lead to musculoskeletal, neurological and vascular damage [36,37]. NVC in HAVS is characterized by degeneration of capillary density, avascular areas, enlarged capillaries, hemorrhages and angiogenesis. The HAVS is a form of secondary RP in which the microvascular damage and the related clinical features, such as local finger blanching, are due to exposition to repetitive vibrations.

A study conducted by Academic Medical Center/University of Amsterdam on volleyball players with ischemic digits of the dominant hand, highlighted the correlation between the digital trauma caused by the collision with the ball and the formation of micro-emboli in digital arteries. The embolic complications of the affected extremity, in combination with pain and ischemia, can lead to microvascular damage [33]. The subjects enrolled in our study didn’t show signs or symptoms resembling RP or HAVS, however our hypothesis is that the repetitive trauma involving fingers could cause damages to microcirculation similar to those presented in patients with HAVS, as we found in the case report of the 17-year-old volleyball player [23].

**Figure 8:**
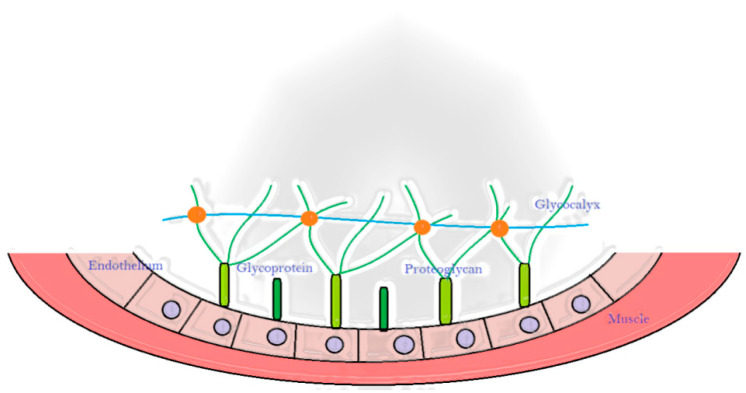
Vascular endothelium is physically sustained by glycocalyx, a carbohydrate-rich layer to which is connected with proteoglycans and glycoproteins in the internal side of the vessels.

The hitting between the ball and the fingers could cause an ischemia/reperfusion condition linked to the alteration of the structure and function of endothelium [38,39]. The vascular endothelium is physically sustained by glycocalyx, a carbohydrate-rich layer to which is connected with proteoglycans and glycoproteins [40] (Figure 8). The endothelial glycocalyx play a central role in the pathophysiology of ischemia/reperfusion-induced tissue damage in particular in response to Radical Oxygen Species produced after a period of decreased flow [41]. Moreover, also the glycocalyx could be damaged by the ischemia/reperfusion, as demonstrated in rat mesenteric venules in which the glycocalyx thickness was significantly reduced, most likely due to shedding of glycosaminoglycan chains [42].

Moreover, we found an early scleroderma pattern in a 29-year-old, healthy setter who had been practicing volleyball for 20 years with weekly 6-h training. It could be hypothesized that role-played, type of action and position of the hands could influence severity and frequency of collision injuries, however, several more volleyball players in each role and with different experiences should be examined [43]. The action carried out by the setter is essentially the pass performed by placing the hands forward above the head to hit the ball with the fingertips of all the fingers of both hands with an intensity that raises the ball.

## 5. Conclusions

This study hypothesizes that volleyball players are prone to damage of the microvasculature probably due to repeated collisions between the fingers and the ball. Any documented capillaroscopic picture represents the starting point for monitoring over time athletes who, aware of the injuries found, can implement the necessary controls and prevention strategies. Early detection of microvascular abnormalities in volleyball players could help to undertake behavioral (functional rest, avoiding sudden cold and rapid changes in temperatures, gloves in cold seasons, finger braces during volleyball practice, hydration of hands, avoid tobacco and caffeine etc.) or pharmacological (e.g., calcium channel blockers, N-acetylcysteine) therapies [44,45].

The goal remains to preserve over time the health of athletes and, where possible, change factors that could affect the good athletic performance. On the basis of the results described, we consider appropriate to carry out screening with NVC in agonistic volleyball players.

The major limitation of the study is the sample small number and secondly, the difference between the number of male and female subjects which makes the sample non-homogeneous. However, this small sample made it possible to quickly conduct this research in order to expand the population in further researches.

Future studies should be conducted on a larger population sample to further investigate the importance of the role played as well as the playing experience on nailfold capillary findings.

## Figures and Tables

**Table 1:** Capillaroscopic parameters and relative characteristics for normal, aspecific and early scleroderma pattern.

Capillaroscopic Parameter	Normal Characteristics	Aspecific Pattern	Early Scleroderma Pattern
**Capillary morphology**	Harpin-like (1), tortuous (2), crossing (3) shape with convex head	Not (1), (2), (3) or non-convex head	Absence of abnormal morphology
**Capillary dimension (µm)**	The diameter of arterial (or afferent) limb can vary from 6 to 19 µm (average value: 11 ± 3 µm). The diameter of the venous (or efferent) limb is generally greater, 8–20 µm (average value: 12 ± 3 µm)	Ectasia of the efferent tract of loops between 20–50 µm	few giant capillaries >50 µm
**Capillary density**	Normal range (≥7, on average between 9 and 14)	Within normal range or lowered density	Within normal range (≥7) No evident loss of capillaries
**Capillary blood flow**	Normally dynamic, no stasis or thrombosis	Granular flow	Irregular flow
**Hemorrhages**	No	Yes/No	Yes/No

**Table 2:** Subjects characteristics and capillaroscopic findings.

Subject	Age	Role	Age of Playing	Training per Week	Match per Week	Capillary Density	Capillary Dimension	Abnormal Morfology	Haemorrhages	Flux	Pattern
**M1**	25	Middle Hitter	10	5	1	↓	20–50	Yes	Yes	Granular	Aspecific
**M2**	23	Middle Hitter	10	5	1	≥7	20–50	Yes	No	Granular	Aspecific
**M3**	25	Libero	18	5	1	≥7	20–50	Yes	No	Granular	Aspecific
**M4**	20	Setter	5	5	1	≥7	Normal	No	No	Normal	Normal
**M5**	26	Opposite Hitter	15	5	1	≥7	20–50	Yes	No	Granular	Aspecific
**M6**	24	Opposite Hitter	11	5	1	≥7	Normal	No	No	Normal	Normal
**M7**	24	Opposite Hitter	11	5	1	≥7	Normal	Yes	No	Normal	Aspecific
**M8**	26					≥7	Normal	No	No	Normal	Normal
**M9**	22					≥7	Normal	No	No	Normal	Normal
**M10**	21					≥7	Normal	No	No	Normal	Normal
**M11**	26					≥7	Normal	No	No	Normal	Normal
**M12**	24					≥7	Normal	No	No	Normal	Normal
**M13**	25					≥7	Normal	No	No	Normal	Normal
**M14**	21					≥7	Normal	No	No	Normal	Normal
**F1**	21	Middle Hitter	13	3	1	≥7	20–50	Yes	No	Granular	Aspecific
**F2**	26	Libero	10	3	1	≥7	20–50	Yes	No	Granular	Aspecific
**F3**	28	Middle Hitter	5	3	1	≥7	20–50	Yes	Yes	Granular	Aspecific
**F4**	26	Opposite Hitter	7	3	1	≥7	20–50	Yes	Yes	Granular	Aspecific
**F5**	29	Setter	20	3	1	≥7	>50 (giant)	No	Yes	Granular	Early Scleroderma Pattern
**F6**	24	Middle Hitter	10	3	1	≥7	20–50	Yes	Yes	Granular	Aspecific
**F7**	29	Libero	15	3	1	≥7	20–50	Yes	No	Granular	Aspecific
**F8**	27	Middle Hitter	4	3	1	≥7	20–50	Yes	No	Granular	Aspecific
**F9**	27	Middle Hitter	12	3	1	Normal	Normal	No	No	Normal	Normal
**F10**	28	Middle Hitter	5	3	1	≥7	20–50	Yes	No	Granular	Aspecific
**F11**	30	Libero	15	4	1	≥7	20–50	Yes	No	Granular	Aspecific
**F12**	32	Opposite Hitter	17	3	1	≥7	20–50	Yes	No	Normal	Aspecific
**F13**	29					≥7	Normal	No	No	Normal	Normal
**F14**	27					≥7	Normal	No	No	Normal	Normal
**F15**	30					≥7	Normal	No	No	Normal	Normal
**F16**	21					≥7	Normal	No	No	Normal	Normal
**F17**	23					≥7	Normal	No	No	Normal	Normal
**F18**	26					≥7	Normal	No	No	Normal	Normal
**F19**	27					≥7	Normal	No	No	Normal	Normal
**F20**	25					≥7	Normal	No	No	Normal	Normal
**F21**	25					≥7	Normal	No	No	Normal	Normal
**F22**	23					≥7	Normal	No	No	Normal	Normal
**F23**	29					≥7	Normal	No	No	Normal	Normal
**F24**	21					≥7	Normal	No	No	Normal	Normal

## Data Availability

The data that support the findings of this study are available on request from the corresponding author.
